# A Context-Dependent Role for αv Integrins in Regulatory T Cell Accumulation at Sites of Inflammation

**DOI:** 10.3389/fimmu.2018.00264

**Published:** 2018-02-26

**Authors:** Iris Mair, Stephanie E. J. Zandee, Iqbal S. Toor, Louise Saul, Rhoanne C. McPherson, Melanie D. Leech, Danielle J. Smyth, Richard A. O’Connor, Neil C. Henderson, Stephen M. Anderton

**Affiliations:** ^1^MRC Centre for Inflammation Research, Centre for Multiple Sclerosis Research, BHF Centre for Cardiovascular Science, and Centre for Immunity Infection and Evolution, University of Edinburgh, Edinburgh, United Kingdom

**Keywords:** integrin αv, Foxp3, regulatory T cell, experimental autoimmune encephalomyelitis, colitis, autoimmune disease, inflammation, resolution of inflammation

## Abstract

Several inflammatory diseases including multiple sclerosis and inflammatory bowel disease have been associated with dysfunctional and/or reduced numbers of Foxp3^+^ regulatory T cells (Treg). While numerous mechanisms of action have been discovered by which Treg can exert their function, disease-specific Treg requirements remain largely unknown. We found that the integrin αv, which can pair with several β subunits including β8, is highly upregulated in Treg at sites of inflammation. Using mice that lacked αv expression or β8 expression specifically in Treg, we demonstrate that there was no deficit in Treg accumulation in the central nervous system during experimental autoimmune encephalomyelitis and no difference in the resolution of disease compared to control mice. In contrast, during a curative T cell transfer model of colitis, Treg lacking all αv integrins were found at reduced proportions and numbers in the inflamed gut. This led to a quantitative impairment in the ability of αv-deficient Treg to reverse disease when Treg numbers in the inflamed colon were below a threshold. Increase of the number of curative Treg injected was able to rescue this phenotype, indicating that αv integrins were not required for the immunosuppressive function of Treg *per se*. In accordance with this, αv deficiency did not impact on the capacity of Treg to suppress proliferation of naive conventional T cells *in vitro* as well as *in vivo*. These observations demonstrate that despite the general upregulation of αv integrins in Treg at sites of inflammation, they are relevant for adequate Treg accumulation only in specific disease settings. The understanding of disease-specific mechanisms of action by Treg has clear implications for Treg-targeted therapies.

## Introduction

Regulatory T cells (Treg) are a major target in the search for novel therapies for immune-mediated diseases—both through pharmacological manipulation and cell-based therapies (1, 2). They display a plethora of mechanisms by which they can mediate immune suppression, including cytolysis, modulation of antigen presenting cells, metabolic disruption, and secretion of inhibitory cytokines such as TGF-β, IL-10, and IL-35 (3). Treg are responsible for the inhibition of inadequate T cell activation in secondary lymphoid organs, but also migrate to inflamed sites to control ongoing inflammation. In order to optimally target Treg, it is important to understand how Treg are recruited to sites of inflammation and which suppressive mechanisms they deploy, which may well be dictated in organ- or disease-specific manners (4–9).

Most circulating Treg display a phenotype resembling that of naive conventional T cells and have been coined central Treg (10). Central Treg are thought to be important in preventing the activation of autoreactive T cells by antigen-presenting cells in secondary lymphoid organs (11). However, a small fraction of circulating Treg displays an activated phenotype and shows enhanced migration through non-lymphoid tissue. The homeostatic requirements of these so-called effector Treg are poorly understood, but at least some populations appear to be IL-2 independent (10, 12, 13). The surface expression of chemokine receptors (10, 14–16), as well as integrins such as αE (17, 18), αL (19), and β7 (5) has been shown to define Treg migration to specific sites.

Integrins, consisting of an α and a β subunit, are important players in cell adhesive functions and motility, but are also involved in intracellular signaling modulating cell survival, proliferation and differentiation (20). αv integrins (αvβ1, αvβ3, αvβ5, αvβ6, αvβ8) all recognize the RGD tripeptide sequence found in several components of the extracellular matrix as well as latent TGF-β (21, 22) and have recently been associated with important functions in several immune cells. αvβ1 and αvβ3 have been linked to interstitial migration of CD4^+^ effector T cells (23) and αvβ8 has been shown to be the key driver for TGF-β activation by CD103^+^ dendritic cells (DCs) and thereby immune homeostasis in the gut (24–26). Recently, the integrin αvβ8 has also been implicated in activation of latent TGF-β by Treg (27, 28). However, their common binding site would suggest that redundancy is highly likely between αv integrins.

On this point, the contribution of αv integrins other than αvβ8 to Treg function in homeostatic or inflammatory settings is unknown. Using conditional knockout mice lacking either all αv integrins or only the integrin αvβ8, we investigated the role of αv integrins in Treg function under inflammatory conditions. We found that αv integrins (but not specifically αvβ8) were required for efficient Treg accumulation in the inflamed intestine and resolution of inflammation, but had no role in Treg activity during central nervous system (CNS) inflammation.

## Materials and Methods

### Mice, Antigens, and Tissue Culture Medium

All mice used were on the I-A^b^ background. Rag1^−/−^ mice and congenically identifiable (CD45.1) OT-II transgenic mice with a TCR reactive toward ovalbumin peptide 323–339 (pOVA) (29) were maintained at the University of Edinburgh. Foxp3^tm4(YFP/cre)Ayr^ mice (30) were kindly provided by Dr. A. Rudensky. *Itgav*^fl/fl^ mice (25) were crossed with Foxp3^tm4(YFP/cre)Ayr^ mice to generate conditional Foxp3-αv^−/−^ mice. Similarly, *Itgb8*^fl/fl^ mice (31) were crossed with Foxp3^tm4(YFP/cre)Ayr^ mice to generate conditional Foxp3-β8^−/−^ mice. Conditional knockout mice were screened by PCR assessing the presence of the floxed αv or β8 gene, respectively, and the presence of the CRE gene. These lines expressed YFP under control of the Foxp3 promoter. All mice were bred under specific pathogen-free conditions at the University of Edinburgh. All experiments were approved by the University of Edinburgh Ethical Review Committee and were performed in accordance with UK legislation. The pOVA peptide and the 35–55 peptide of mouse myelin oligodendrocyte glycoprotein (pMOG) were obtained from Cambridge Research Biochemicals (Teesside, UK). Tissue culture medium (RPMI 1640 medium) was supplemented with 2 mM l-glutamine, 100 U/ml penicillin, 100 µg/ml streptomycin, and 50 µM 2-ME (all from Gibco, Paisley, UK) plus 10% FCS (Labtech, East Sussex, UK).

### Cell Purification and Culture

CD4^+^ T cells were purified by magnetic cell sorting (Miltenyi Biotec, Germany) prior to surface staining and sorting by FACS. CD4^+^YFP^-^CD62L^hi^ naive T cells from Foxp3^tm4(YFP/cre)Ayr^ mice were used as responder T cells for *in vitro* suppression assays as well as disease-inducing cells in the T cell transfer colitis models. CD4^+^YFP^-^CD62L^hi^ naive T cells from Foxp3^tm4(YFP/cre)Ayr^, Foxp3-αv^−/−^ or Foxp3-β8^−/−^ mice were also used as starting populations for induced Treg (iTreg) generation as previously described (32). CD4^+^YFP^+^ nTreg were isolated from Foxp3^tm4(YFP/cre)Ayr^, Foxp3-αv^−/−^ or Foxp3-β8^−/−^ mice and tested for their suppressive capacity in *in vitro* suppression assays or in T cell transfer colitis models. *In vitro* stimulation was provided by anti-CD3e (clone 145.2C11; eBioscience, Hatfield, UK) plus anti-CD28 (clone 37.51; eBioscience) at 2 µg/ml each. Cell culture supernatants were tested for IL-10 concentrations using a mouse IL-10 ELISA kit according to manufacturer’s instructions (eBioscience).

### *In Vitro* Suppression Assay

Suppression assays were performed by culturing CD4^+^YFP^-^CD62L^hi^ naive responder T cells (2 × 10^4^/well) for 96 h with increasing numbers of CD4^+^YFP^+^ nTreg from Foxp3 reporter mouse lines (0–2 × 10^4^/well) in the presence of irradiated (30 Gray) splenic APCs and 1 µg/ml anti-CD3e. For the last 16 h of culture, 0.25 μCi of ^3^H-thymidine (Amersham Biosciences, Amersham, UK) was added to each well and incorporation determined as mean counts per minute (cpm) using a β-scintillation counter (Wallac, Turku, Finland).

### *In Vivo* Priming

Host mice (CD45.2) received 2 × 10^6^ CD4^+^ OT-II T cells (CD45.1) i.v. 1 day prior to immunization with 20 µg pOVA emulsified in complete Freund’s adjuvant (CFA, containing 200 µg heat-killed *Mycobacterium tuberculosis* H37Ra) (Sigma) in a final volume of 100 µl. Seven days after immunization, spleens and draining lymph nodes were isolated for flow cytometric analysis.

### Induction of Experimental Autoimmune Encephalomyelitis (EAE)

Mice were immunized by subcutaneous injection of 100 µg pMOG, emulsified in CFA in a final volume of 100 µl. Mice received 200–250 ng of Pertussis toxin in 500 µl PBS i.p. on the same day as immunization and 2 days later (33). Clinical signs of EAE were scored daily using the following scoring system; 0, no signs; 1, flaccid tail; 2, impaired righting reflex and/or gait; 2.5, impaired gait including notable but intermittent dragging of feet; 3, partial hind limb paralysis; 4, total hind limb paralysis; 5, hind limb paralysis with partial forelimb paralysis; 6, moribund or dead. Mice were sacrificed by CO_2_ asphyxiation at the latest 30 days after disease induction, or at an earlier time point to isolate organs for analysis. Assessment of CNS (brain and spinal cord) immune cells was conducted as described previously (34).

### T Cell Transfer Colitis

Preventive model of T cell transfer colitis; RAG1^−/−^ mice were injected i.v. with PBS or 5 × 10^5^ naive wild-type (WT) CD4^+^ T cells (CD4^+^CD62L^hi^YFP^-^) from Foxp3^tm4(YFP/cre)Ayr^ mice in the presence or absence of 1.5 × 10^5^ nTreg (CD4^+^YFP^+^) from Foxp3^tm4(YFP/cre)Ayr^ or Foxp3-αv^−/−^ mice in a total volume of 200 µl.

Curative model of T cell transfer colitis; RAG1^−/−^ mice were injected i.v. with PBS or 5 × 10^5^ naive WT CD4^+^ T cells (CD4^+^CD62L^hi^YFP^-^) from Foxp3^tm4(YFP/cre)Ayr^ mice, followed by i.v. injection of 2.5–8 × 10^5^ nTreg (CD4^+^YFP^+^) from the indicated Foxp3 reporter mice 21 days later. Mice were monitored daily and weighed three times a week until cull at 6–9 weeks after naive T cell transfer for analysis of spleen, mesenteric lymph nodes (MLNs), and colons. One half of the colonic tissue was used to isolate lymphoid cells from the lamina propria (LP). Briefly, the intestinal epithelial layer was removed by incubation in HBSS 2 mM EDTA for 30 min, and the remaining tissue digested with 1.25 mg/ml collagenase-4 (Worthington) and 30 µg/ml DNase-1 (Roche) in culture medium and disaggregated with a gentle MACS dissociator (Miltenyi). Retrieved lymphoid cells were stained and analyzed by flow cytometry. The other half of colonic tissue was processed for staining by hematoxylin and eosin and assessed for colitis severity in a blinded fashion using the following scoring system for a maximum of 11 points; mucosal infiltration (0-3); submucosal infiltration (0-3); crypt loss (0-3); crypt abscesses (0-2).

### Antibodies and FACS Analysis

Cells were stained with the following antibodies (all from eBioscience, except where stated); anti-CD4-BV650 (BioLegend, San Diego, CA, USA), anti-Foxp3-(ef450/FITC), anti-CD11b-AF700 (Biolegend), anti-αv-PE, anti-CD44-APC-Cy7, anti-CD62L-PerCP-Cy5.5, anti-Ki-67-PE-Cy7, anti-KLRG1-ef450, anti-CD45.1-APC, anti-CD51-PE, anti-GM-CSF-PE, anti-IFN-γ-APC, anti-IL-17-PerCPCy5.5. Rat IgG2a and rat IgG1, conjugated to respective fluorophores, were used as isotype control antibodies. For intracellular cytokine staining, cells were re-stimulated *ex vivo* with 20 µg/ml pOVA overnight, and Brefeldin A added for the last 4 h of incubation. Samples were stained with a fixable viability marker (conjugated with eFluor455, eBioscience) prior to surface staining. For subsequent intracellular antigen staining, samples were washed once in FACS buffer (PBS, 2% FCS, 0.01% NaN_3_) and then processed according to manufacturer’s instructions (eBioscience for transcription factors, BD for intracellular cytokines). Flow cytometric data were acquired using a BD LSR Fortessa cell analyzer (BD) and data analyzed using FlowJo software (Treestar version 3.2.1, Ashland, OR, USA).

### Immunohistochemistry

Paraffin-embedded longitudinal sections of the gut were cleared and rehydrated in xylene and graded alcohols, followed by antigen retrieval through pressure boiling in citrate buffer (Vector, Burlingame, CA, USA). Endogenous peroxidase activity was blocked with 3% H_2_O_2_ (Fisher Scientific) in dH_2_O, 10% normal goat serum (Biosera, Boussens, France) in PBS was used to block unspecific binding sites, and endogenous avidin/biotin was blocked using an avidin-biotin blocking kit according to manufacturer’s instructions (Vector). Samples were stained with biotinylated rat anti-mouse Foxp3 antibody (eBioscience, clone FJK-16s) and purified rabbit anti-mouse CD3 antibody (Abcam, polyclonal). Samples were then incubated with streptavidin-coupled alkaline phosphatase (Vector) for 1 h at room temperature, washed, and Vector Blue substrate (SK-5300, Vector) added according to manufacturer’s instructions. Samples were subsequently incubated with horseradish peroxidase-coupled goat anti-rabbit antibody, washed, and DAB working solution added according to manufacturer’s instructions (Vector). Slides were mounted in Permafluor mounting medium (Thermo Scientific) prior to digitalization with an Axioscan.Z1 slide scanner (Carl Zeiss).

### Statistical Analysis

Statistical analyses were performed using GraphPad Prism software (San Diego, CA, USA). In order to verify that data were normally distributed, Shapiro–Wilk normality tests were conducted. In experiments where data followed a Gaussian distribution, significance was tested using an unpaired Student’s *t*-test when comparing two experimental groups, and a one-way ANOVA with Bonferroni’s *post hoc* test when comparing three or more experimental groups. When *n* numbers were not sufficient to test for normality, or when data proved not to be normally distributed, the non-parametric Mann–Whitney test (comparing two groups) or the non-parametric Kruskal–Wallis test with Dunn’s *post hoc* test (three or more groups) were applied. Due to the ranked nature of EAE disease scores and disease length, the non-parametric Kruskal–Wallis test with Dunn’s *post hoc* test was used to determine differences between three or more groups. When examining the effect of two factors on the experimental outcome, a two-way ANOVA was applied with Bonferroni’s *post hoc* test where applicable. Data were considered significantly different with *p*-values of <0.05 (*p < 0.05, **p < 0.01, and ***p < 0.001).

## Results

### Treg at Sites of Inflammation Express High Levels of the Integrin αv

**Figure 1:**
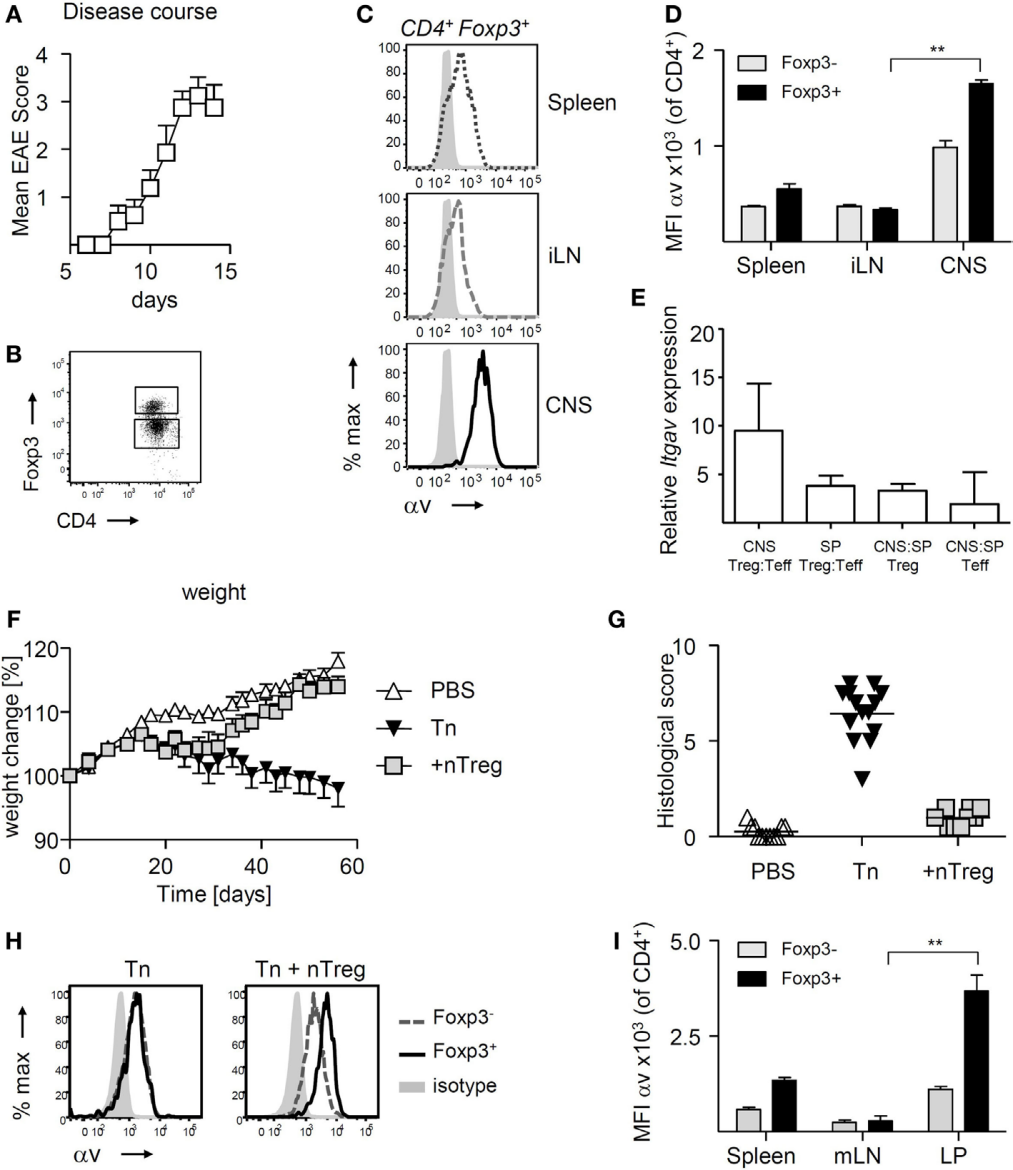
αv is highly expressed on regulatory T cells (Treg) at sites of inflammation experimental autoimmune encephalomyelitis (EAE) was induced in Foxp3^tm4(YFP/cre)Ayr^ mice by immunization with 35–55 peptide of mouse myelin oligodendrocyte glycoprotein (pMOG), and spleen, inguinal lymph nodes (iLN), and central nervous system (CNS) harvested shortly after peak of disease (*n* = 8, pooled from two experiments). **(A)** Mean clinical scores + SEM. **(B)** Representative dot plot for gating of CNS Foxp3^+^ and Foxp3^−^ CD4^+^ T cells. **(C)** Representative plots of αv expression on Treg retrieved from different organs. **(D)** αv expression levels on Treg in comparison to conventional T cells in different organs (*n* = 4, representative of two experiments). **(E)** Relative mRNA expression levels of αv in Treg and conventional T cells in CNS and spleen of EAE mice. **(F)** Colitis was induced by injection of naive T cells (Tn) into RAG1^−/−^ mice and curative CD4^+^ Foxp3^+^ T cells (nTreg) were injected 21 days later (*n* = 10–14 mice per group, pooled from two experiments). Spleen, mesenteric lymph nodes (mLNs) and colon lamina propria (LP) were harvested 8 weeks after disease induction. **(G)** Histological colitis score. **(H)** Representative histograms showing αv expression on Foxp3^+^ and Foxp3^−^ CD4^+^ T cells in the LP of colitis mice. **(I)** αv expression levels on Treg in comparison to conventional T cells in different organs (*n* = 4, representative of two experiments).

Experimental autoimmune encephalomyelitis is a useful experimental model to compare the function and behavior of CD4^+^ T cells, including Treg, retrieved from the site of autoimmune inflammation (in this case the CNS) versus those from the secondary lymphoid organs of the same mouse. We have previously documented that Treg retrieved from the inflamed CNS display a suppressive potency that is markedly superior to that of their splenic counterparts (35). Here, we sought to understand any causative association between this elevated Treg function and their expression of αv integrins. Treg retrieved from the inflamed CNS shortly after peak of disease (Figures 1A,B) exhibited markedly higher surface αv expression levels compared to Treg in the spleen and inguinal lymph nodes (iLNs) (Figures 1C,D). Treg surface αv expression levels were also higher compared to effector T cells, in both spleen and CNS (Figure 1D). These differences were confirmed at the mRNA level using qPCR of sorted CD4^+^ T cell populations (Figure 1E).

To understand whether increased αv expression was a more general feature of Treg exposed to inflammation, we investigated αv expression patterns in a curative T cell transfer model of colitis. Treg were injected into RAG1^−/−^ mice 3 weeks after disease induction and colon LP, mLNs, and spleen harvested 6 weeks later (Figures 1F,G). Following an initial weight loss typical for T cell induced colitis, mice treated with Treg started regaining weight about 1 week post-Treg injection, indicative of the immunosuppressive activity of the injected Treg. Again, Treg found in the colon LP expressed αv at markedly higher levels than their splenic and mLN counterparts, as well as their Foxp3^−^ counterparts (Figures 1H,I), suggesting that Treg at sites of inflammation may generally exhibit enhanced αv expression levels.

### Treg Homeostasis Is Not Dependent on αv Integrins

**Figure 2:**
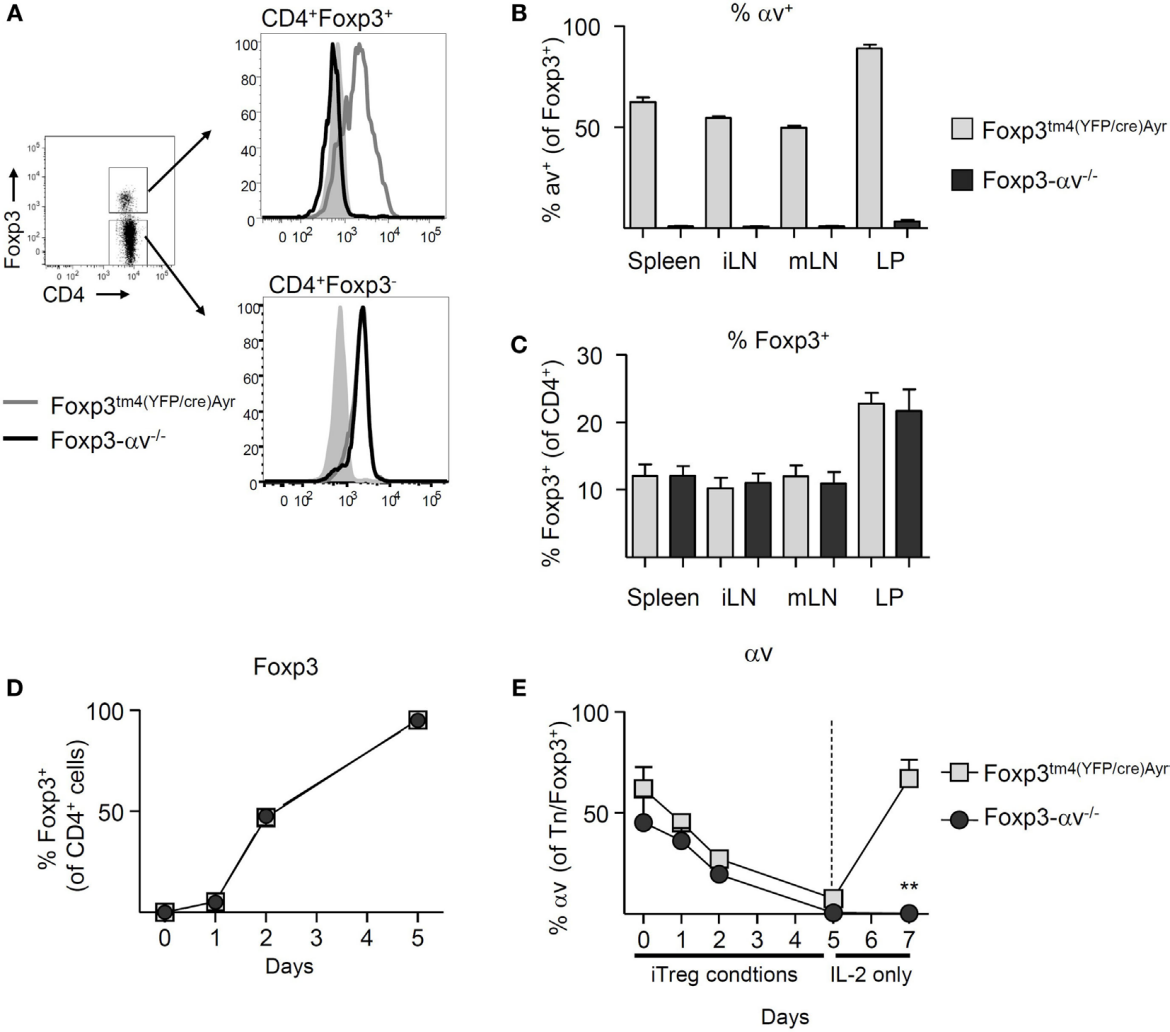
Basic characterization of regulatory T cells (Treg) from Foxp3-αv^−/−^ mice. **(A)** Representative histograms of αv expression in Foxp3^+^ and Foxp3^−^ CD4^+^ T cells in unmanipulated Foxp3^tm4(YFP/cre)Ayr^ and Foxp3-αv^−/−^ mice. **(B)** Proportion of αv^+^ Treg in WT and Foxp3-αv^−/−^ mice (*n* = 7–10, pooled from three experiments). **(C)** Proportion of Foxp3^+^ Treg in Foxp3^tm4(YFP/cre)Ayr^ and Foxp3-αv^−/−^ mice (*n* = 6–7 mice per group, pooled from three experiments). Naive CD4^+^ T cells from Foxp3^tm4(YFP/cre)Ayr^ and Foxp3-αv^−/−^ mice were cultured under iTreg generating conditions and **(D)** Foxp3 expression assessed over time (*n* = 6 mice per group, pooled from two experiments). iTreg were rested in IL-2 for 2 days and **(E)** proportion of Foxp3^+^ iTreg (naive T cells on d0) expressing αv determined over time.

In order to test whether αv integrins are required by Treg to maintain immune homeostasis, we generated conditional Foxp3-αv^−/−^ mice lacking αv specifically in Foxp3^+^ cells by crossing Foxp3^tm4(YFP/cre)Ayr^ mice (30) with *Itgav*^fl/fl^ mice (25). Both naive CD4^+^ T cells and Treg from Foxp3^tm4(YFP/cre)Ayr^ control mice expressed αv (Figure 2A). As expected, surface αv expression was absent specifically among Treg from Foxp3-αv^−/−^ mice (Figures 2A,B). Importantly, proportions of Treg in spleen, lymph nodes and colon LP remained unaltered in the absence of Treg αv integrins (Figure 2C), indicating that αv integrins are not involved in Treg maturation and homeostasis. Treg from Foxp3-αv^−/−^ mice and Foxp3^tm4(YFP/cre)Ayr^ control mice displayed comparable CD62L and CD44 expression in the steady state, indicating unaltered activation status in the absence of αv integrins (Figure S1A in Supplementary Material). Similarly, Ki-67 levels were indistinguishable between control and αv^−/−^ nTreg, suggesting no alteration in cell cycle progression and hence proliferative potential in these cells (Figure S1B in Supplementary Material). Foxp3 expression levels within the Foxp3^+^ Treg pool were also comparable between control and Foxp3-αv^−/−^ mice (Figure S1C in Supplementary Material).

In order to verify that the deletion of the *Itgav* gene occurred upon induction of Foxp3, iTreg were generated from naive CD4^+^ T cells of Foxp3^tm4(YFP/cre)Ayr^ control and Foxp3-αv^−/−^ mice for 5 days. >50% of naive CD4^+^ T cells in both groups expressed αv at the start of culture. At the end of culture, >95% of the cells in both groups expressed Foxp3 (Figure 2D). Surprisingly, αv expression was downregulated on all cells over the course of 5 days in iTreg culture (Figure 2E). However, upon replenishment of culture media without TGF-β for 2 days, control iTreg readily recovered αv expression, while Foxp3-αv^−/−^ iTreg were unable to do so (Figure 2E). This demonstrated that the ability to express αv in CD4^+^ T cells was lost upon Foxp3 induction in these conditional knockout mice.

Considering the marked impact of iTreg culture conditions on αv expression in these cells, this phenomenon was further examined. Stimulation of naive T cells, in the absence of active TGF-β, did not result in a loss of αv expression (Figure S1D in Supplementary Material), suggesting that the presence of active TGF-β during culture caused downregulation of αv. Since stimulation of naive T cells in the presence of TGF-β led to reduced αv expression even in CD4^+^ T cells not expressing Foxp3 within 5 days (Figure S1E in Supplementary Material), downregulation of αv was unlikely a secondary effect of Foxp3 induction. A similar effect was observed in nTreg, whereby nTreg stimulated in the presence of active TGF-β for 3 days displayed significantly lower levels of αv compared to nTreg stimulated in the absence of active TGF-β (Figure S1F in Supplementary Material). Loss of surface αv through the presence of TGF-β was partially prevented by the addition of a TGF-β type I receptor (ALK5) kinase inhibitor, supporting the idea that TGF-β signaling itself led to αv downregulation.

### Treg Do Not Require αv Expression in Order to Suppress the Activation and Proliferation of Conventional T Cells

**Figure 3:**
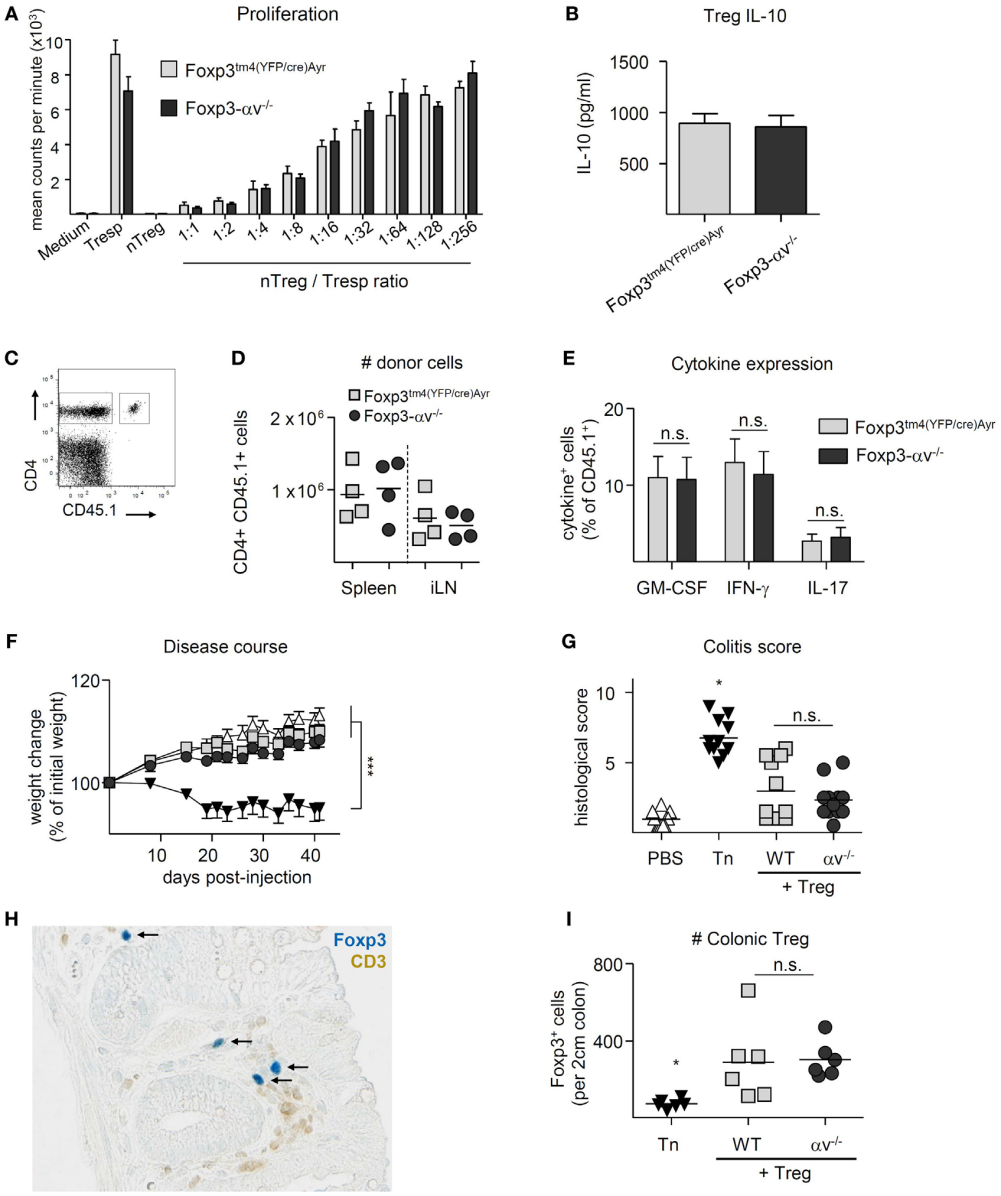
αv-deficient regulatory T cells (Treg) can suppress responder T cell proliferation *in vitro* and *in vivo*. **(A)** Varied numbers of Foxp3^tm4(YFP/cre)Ayr^ or Foxp3-αv^−/−^ nTreg were cocultured with a fixed number of Foxp3^tm4(YFP/cre)Ayr^ naive T cells in the presence of irradiated antigen presenting cells and soluble anti-CD3 for 4 days. Cell proliferation was measured by ^3^H-thymidine incorporation in the last 18 h of culture (*n* = 2–3 mice per group). **(B)** Foxp3^tm4(YFP/cre)Ayr^ or Foxp3-αv^−/−^ nTreg were cultured with mouse T-Activator Dynabeads at a 1:1 ratio for 3 days in the presence of 1,000 U/ml IL-2, and IL-10 concentrations measured in the culture supernatant by ELISA (*n* = 6, pooled from two experiments). Foxp3^tm4(YFP/cre)Ayr^ and Foxp3-αv^−/−^ mice were immunized with pOVA in CFA 1 day after transfer of 2 × 10^6^ CD4^+^ OT-II responder cells (CD45.1), and organs harvested 7 days later. **(C)** Gating strategy for host (CD45.1^−^) and donor (CD45.1^+^) CD4^+^ T cells and **(D)** total numbers of donor cells retrieved from spleen and inguinal lymph nodes (iLNs). **(E)** Splenocytes were restimulated with 20 µg/ml pOVA overnight and cytokine expression analyzed by intracellular cytokine staining. **(F)** Colitis was induced by injection of naive T cells (Tn) into RAG1^−/−^ mice in the presence or absence of Foxp3^tm4(YFP/cre)Ayr^ or Foxp3-αv^−/−^ nTreg and weight assessed over time (*n* = 11–12 mice per group, pooled from two experiments). **(G)** Histological colitis score 6 weeks after disease induction (range = 0–11). **(H)** Colon sections were stained with anti-Foxp3 (see black arrows) and anti-CD3 antibodies and visualized with Vector Blue substrate and DAB, respectively. **(I)** Numbers of Foxp3^+^ cells were counted in a 2 cm long longitudinal section of the colon, starting 2 cm proximal of the anus (*n* = 6 per group).

Conventional CD4^+^ T cells in spleen, iLN, mLN, and colon LP of Foxp3^tm4(YFP/cre)Ayr^ control mice and Foxp3-αv^−/−^ mice displayed a similar activation status and proliferative activity (Figures S2A,B in Supplementary Material), indicating normal Treg activity in the absence of αv integrins under steady state. Indeed, Foxp3-αv^−/−^ mice did not develop a spontaneous inflammatory disorder when left to age up to 10 months (data not shown). Consistent with this, no differences were found in the ability of nTreg from αv^−/−^ or Foxp3^tm4(YFP/cre)Ayr^ mice to inhibit responder T cell proliferation using an *in vitro* suppression assay (Figure 3A). Likewise, iTreg generated from Foxp3-αv^−/−^ mice showed no impairment in suppressive activity *in vitro* (data not shown). The secretion of IL-10, a major Treg-derived immunosuppressive cytokine, was also not impaired in αv^−/−^ Treg upon *in vitro* stimulation (Figure 3B).

To test Treg suppressive function *in vivo*, Foxp3-αv^−/−^ and Foxp3^tm4(YFP/cre)Ayr^ control mice were seeded with CD4^+^CD45.1^+^ T cells from OT-II mice prior to immunization with pOVA. Proportions and total numbers of CD45.1^+^ donor cells that had subsequently accumulated in the spleen or draining lymph nodes did not differ between Foxp3-αv^−/−^ and control mice (Figures 3C,D, data not shown). Also, no difference was observed in donor cell activation and proliferation status (Figures S3A,B in Supplementary Material), and pro-inflammatory cytokine expression upon re-stimulation with cognate antigen *ex vivo* was comparable between groups (Figure 3E). Similarly, αv integrins were not required for Treg suppressive activity in a preventive model of T cell transfer colitis. αv^−/−^ Treg co-injected with disease-inducing naive CD4^+^ T cells from Foxp3^tm4(YFP/cre)Ayr^ mice were as capable of preventing pathological changes of the colon and associated weight loss as control Treg (Figures 3F,G). Notably, immunohistochemical staining of Foxp3^+^ cells demonstrated comparable numbers of control and αv^−/−^ Treg in the colon LP of these mice (Figures 3H,I). Collectively, these data indicate that Treg αv integrins play no critical role in their capacity to limit inflammation initiated by the activities of CD4^+^ T cells.

### αv^−/−^ Treg Can Migrate to the Inflamed CNS and Resolve EAE

**Figure 4:**
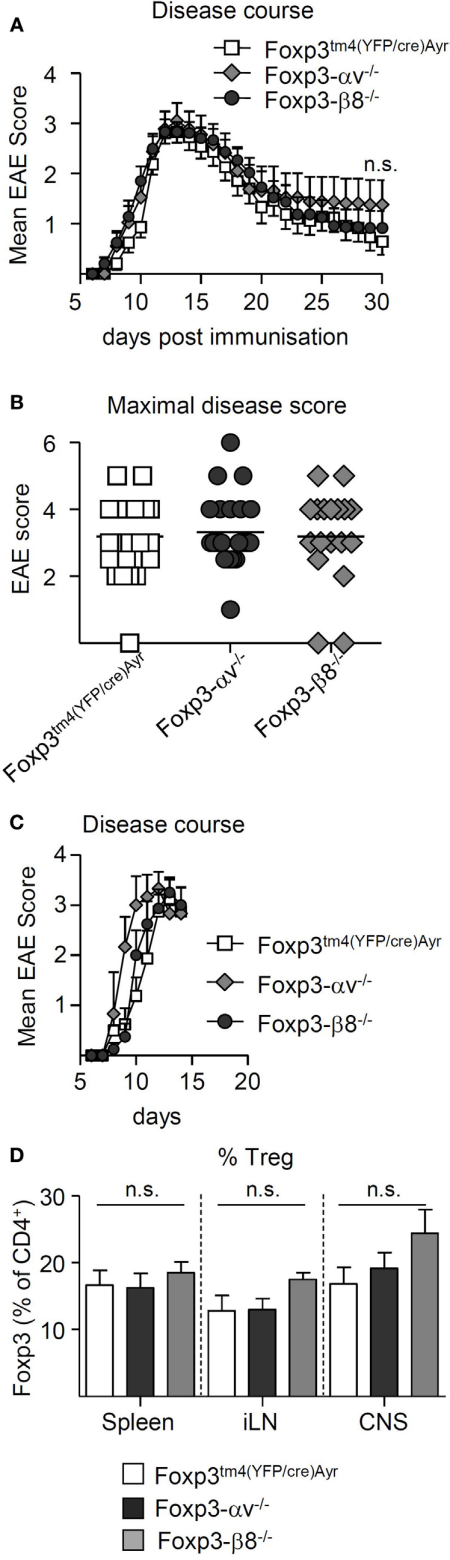
αv-deficient regulatory T cells (Treg) are able to migrate to the inflamed central nervous system (CNS) and resolve experimental autoimmune encephalomyelitis (EAE). EAE was induced in Foxp3^tm4(YFP/cre)Ayr^, Foxp3-αv^−/−^, and Foxp3-β8^−/−^ mice by immunization with pMOG and mice monitored up to 30 days. **(A)** Mean clinical scores ± SEM and **(B)** maximal disease scores (*n* = 18–24 mice per group, pooled from four experiments). EAE was induced as above and spleen, inguinal lymph nodes (iLNs), and CNS harvested shortly after peak of disease (*n* = 3–8, pooled from two experiments). **(C)** Mean clinical scores + SEM. **(D)** Proportion of Treg in indicated organs.

The integrin αvβ8 has been recently proposed to be essential for Treg function at sites of inflammation through the activation of latent TGF-β (28). During EAE, CNS Treg exhibit a highly activated phenotype (34, 35), and Treg are crucial in mediating the resolution of disease (35–37). While αv integrins appeared redundant for Treg homeostasis and Treg-mediated prevention of inflammation, it remained to be determined whether the observed elevated levels of αv on CNS Treg (Figures 1C–E) had a functional consequence for resolution of EAE. The role of αv integrins, and particularly the integrin αvβ8, in Treg function during EAE was explored in Foxp3^tm4(YFP/cre)Ayr^, Foxp3-αv^−/−^ and Foxp3-β8^−/−^ mice, following immunization with pMOG. The clinical courses and mean maximal EAE scores were indistinguishable between all three mouse lines (Figures 4A,B), indicating no requirement for αv integrins in Treg-dependent resolution of EAE. Shortly after peak of disease, αv^−/−^ and β8^−/−^ Treg were found at similar proportions in the CNS as WT Treg (Figures 4C,D), excluding a critical role for αv integrins in Treg migration into the inflamed CNS. CNS Treg in all three mouse lines displayed an effector Treg phenotype, with high CD44 and low CD62L expression, high cell cycle activity and heightened KLRG1 expression, compared to their peripheral counterparts (Figures S4A–C in Supplementary Material).

### Impaired Accumulation of αv^−/−^ Treg in the Inflamed Gut and Quantitative Defect to Cure T Cell-Induced Colitis

Although Treg are critical to the immune processes that ultimately lead to the resolution of EAE, it remains unclear whether this non-redundant influence is exerted solely by those Treg that accumulate in the inflamed CNS, or whether an alternative, earlier effect of restraining pathogenic T cell expansion during the priming stages of EAE in the peripheral lymphoid organs is also required, or even indispensable. To more specifically address the activity of Treg once inflammatory pathology was established, we chose the curative model of T cell transfer colitis, whereby Treg are injected into RAG1^−/−^ after disease onset (38), and control of colonic inflammation can only occur if Treg migrate to the site of inflammation.

**Figure 5:**
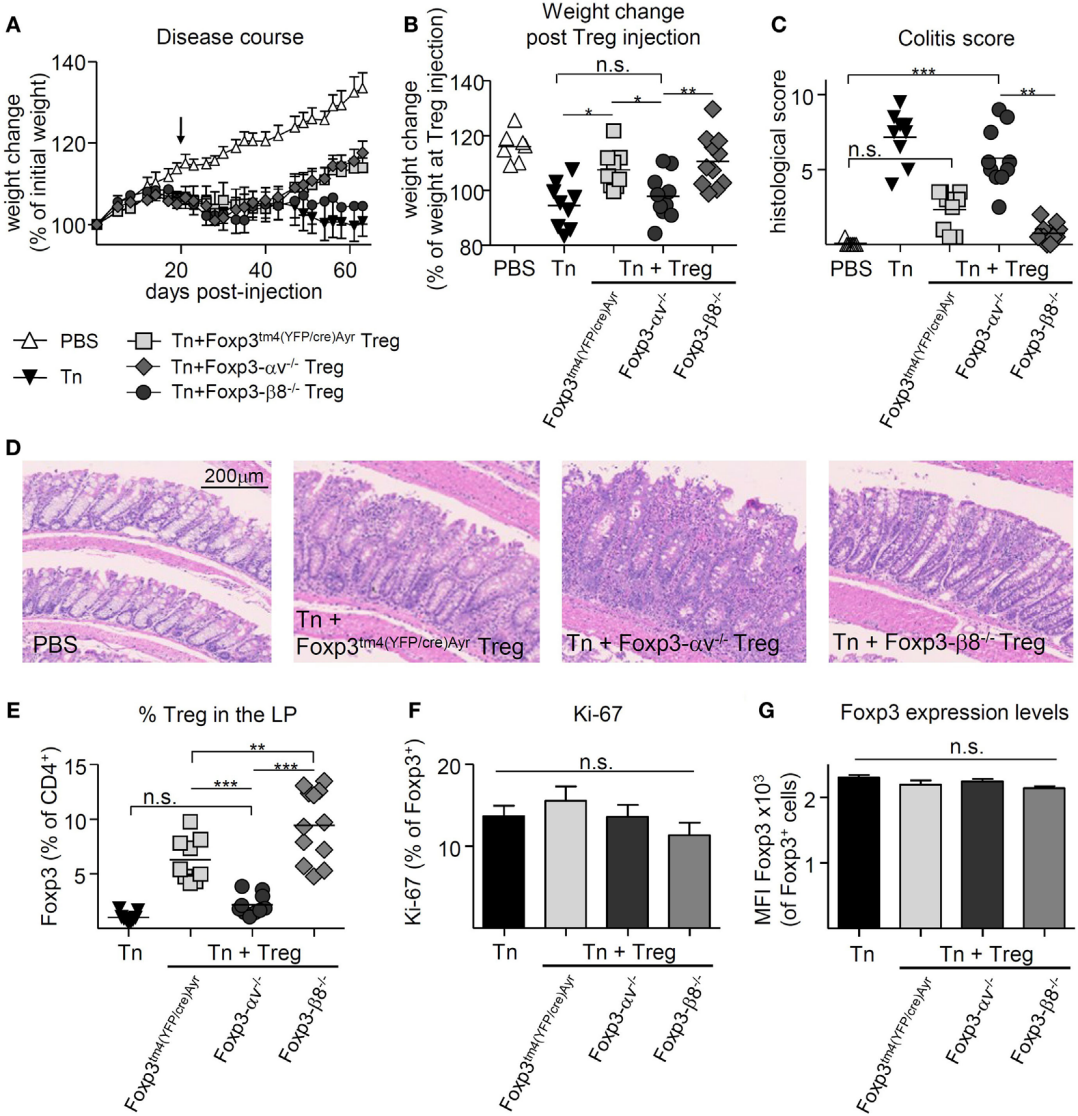
Regulatory T cells (Treg) lacking all αv integrins are unable to cure T cell-induced colitis due to attenuated accumulation in the inflamed gut. Colitis was induced by injection of naive T cells (Tn) into RAG1^−/−^ mice, and 2.5 × 10^5^ nTreg from Foxp3^tm4(YFP/cre)Ayr^, Foxp3-αv^−/−^, or Foxp3-β8^−/−^ mice injected 21 days later (*n* = 7–12 mice per group, pooled from two experiments). Spleen, mesenteric lymph nodes (mLN), and colon lamina propria (LP) were harvested 9 weeks after disease induction. Weight change **(A)** over time and **(B)** at harvest relative to weight at nTreg injection. **(C)** Histological colitis score (range = 0–11) and **(D)** representative H&E-stained sections of the colon LP. Proportion of **(E)** Treg and **(F)** Ki-67^+^ Treg in the colon LP. **(G)** Foxp3 expression levels of Treg in the colon LP (*n* = 4–6, representative of two experiments).

**Figure 6:**
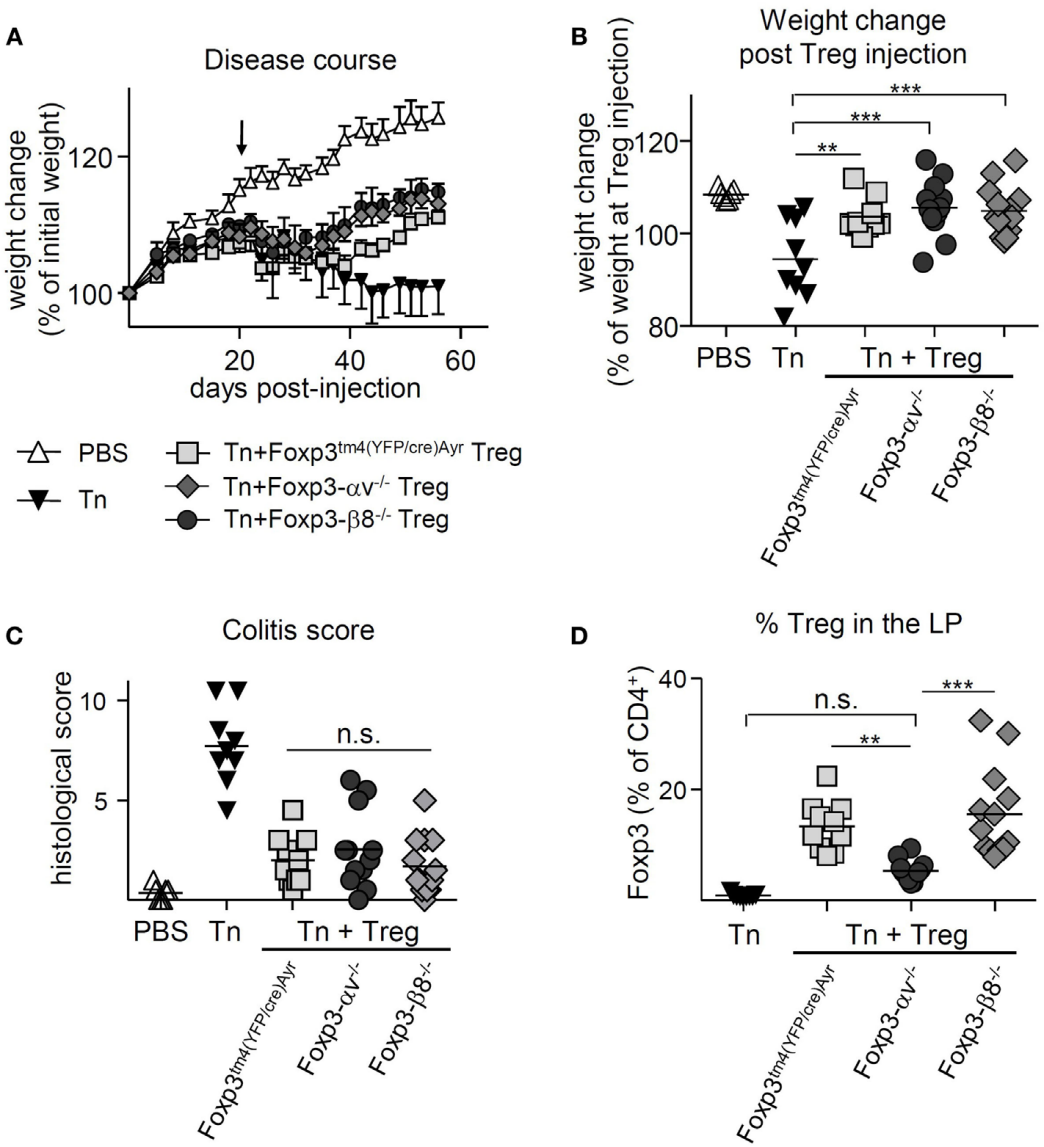
High numbers of injected αv-deficient regulatory T cells (Treg) allow for disease resolution in T cell-induced colitis. Colitis was induced by injection of naive T cells (Tn) into RAG1^−/−^ mice, and 8 × 10^5^ nTreg from Foxp3^tm4(YFP/cre)Ayr^, Foxp3-αv^−/−^, or Foxp3-β8^−/−^ mice injected 21 days later (*n* = 9–13 mice per group, pooled from two experiments). Spleen, mesenteric lymph nodes (mLN), and colon lamina propria (LP) were harvested 9 weeks after disease induction. Weight change **(A)** over time and **(B)** at harvest relative to weight at nTreg injection. **(C)** Histological colitis score (range = 0–11) and **(D)** proportion of Treg in the colon LP.

T cell transfer colitis was induced in RAG1^−/−^ mice, and Foxp3^tm4(YFP/cre)Ayr^, αv^−/−^ or β8^−/−^ Treg were injected at 2.5 × 10^5^ cells per mouse 21 days later. Both the control Treg-treated and the β8^−/−^ Treg-treated mice began regaining weight around 3 weeks after Treg injection (Figure 5A). However, αv^−/−^ Treg-treated mice failed to regain weight (Figure 5A), translating into no weight gain from the time of Treg injection, compared to mice receiving either control or β8^−/−^ Treg (Figure 5B). Consistent with this, histological severity of colitis differed significantly between groups (Figures 5C,D); while mice receiving control Treg and β8^−/−^ Treg had relatively low colitis scores, those receiving αv^−/−^ Treg had disease scores which did not differ from diseased mice that received no Treg treatment. This failure of αv^−/−^ Treg to cure colitis was associated with significantly reduced Treg proportions and numbers in the LP (Figure 5E; Figure S5A in Supplementary Material). In contrast, Treg proportions and numbers in the spleen and mLN were not reduced (Figures S5B-E in Supplementary Material). However, the proportion of Treg expressing Ki-67 as well as Foxp3 expression levels of Treg in the LP did not differ between groups (Figures 5F,G), suggesting that neither reduced Treg proliferation nor Foxp3 instability were the cause of the observed lower αv^−/−^ Treg proportions in the LP. Finally, at a higher number of curative Treg (8 × 10^5^) injected 21 days postdisease induction, control and αv^−/−^ Treg were equally able to reverse disease (Figures 6A–C). While a significantly reduced proportion of αv^−/−^ Treg in the colon LP was still apparent at harvest (Figure 6D), they represented an average 5.4% of the colonic CD4^+^ T cell population versus 2.2% in the first curative colitis experiment. This suggests that a quantitative, rather than qualitative, deficit was responsible for the observed inability of αv^−/−^ Treg to cure colitis at lower Treg injection numbers.

## Discussion

Our findings highlight the tissue- and context-specific requirement for Treg αv integrins in optimal Treg function. αv integrins were not required for Treg to migrate to the inflamed CNS during EAE and resolve disease. Furthermore, αv^−/−^ Treg were able to prevent T cell transfer colitis. However, during a curative T cell transfer model of colitis, αv^−/−^ Treg were detected at significantly lower proportions and numbers in the inflamed gut, and this correlated with an impairment in the ability of αv^−/−^ Treg to resolve colitis compared to WT Treg.

### αv Integrins Are Crucial for Treg Accumulation in the Inflamed Gut

The curative model of T cell transfer colitis is a useful tool to assess whether Treg can migrate to the already established site of inflammation in the periphery and resolve ongoing inflammation. When injecting naive CD4^+^ T cells alone, a small proportion of these cells are converted into Foxp3 expressing peripherally induced Treg in the gut (39). Mice receiving 2.5 × 10^5^ αv^−/−^ Treg showed similar proportions and numbers of Foxp3^+^ cells in the colon LP as control mice that received no Treg injection, associated with a failure to reverse disease. While injection of a higher number (8 × 10^5^) of curative αv^−/−^ Treg resulted in a greater proportion of Treg in the colon LP and the ability to resolve disease, the deficit in Treg accumulation in the colon in the absence of αv compared to WT Treg was still apparent. Integrins have been implicated in the migratory capacity of many cell types (40). However, subsets of CD4^+^ T cells do not share all migratory pathways, with integrin α4 being critical for Th1 cell entry into the CNS during EAE while being redundant for Treg entry (19). Instead, Treg rather depend on αL to cross the blood–brain barrier. Mechanisms of Treg migration may also be tissue-specific, since Treg found in different tissues have a distinct gene expression profile, adapted to the respective environment (4, 41–44). It is therefore not surprising that in a different experimental model of tissue inflammation, namely EAE, αv^−/−^ Treg were found to accumulate at expected levels in the inflamed CNS. Overstreet et al. reported no role for αv integrins in effector T cell migration from lymph nodes into the inflamed skin (23). However, ECM changes during inflammation promoted a role for αv integrins in interstitial movement within the skin, thereby aiding clearance of infection. Since αv integrins are able to recognize several components of the ECM, a role in controlling migration in inflamed versus non-inflamed tissue is conceivable. This is further supported by the observation that αv^−/−^ Treg were found at comparable levels to control Treg in the colon during the preventive model of T cell transfer colitis. In this model, coinjection of Treg and naive CD4^+^ T cells allows for Treg to migrate through and populate uninflamed tissue, unlike in the curative model. This does not exclude a potential role for αv integrins in the survival (10), Foxp3 stability (45), and expansion of Treg (34) in the inflamed colon, which have all been implicated in defining Treg accumulation in other contexts. Unaltered Treg numbers in secondary lymphoid organs and the CNS, in the case of EAE, does not preclude the possibility that αv integrins are required for Treg survival in the inflamed gut, since different Treg subsets have been reported to depend on specific survival signals, such as ICOS in the case of effector Treg (10) and IL-2 in the case of central Treg (46, 47). While we confirmed comparable Foxp3 and Ki-67 levels in colonic Treg, arguing against altered Treg stability and local expansion, respectively, the data reflect only the circumstances of the time point analyzed. However, there is strong evidence that αv^−/−^ Treg have no functional deficit in their immunosuppressive capacity. This is supported by their ability to suppress activation of responder T cells *in vitro* as well as in several *in vivo* models to the same extent as WT Treg, and their equal expression of IL-10 upon activation—a cytokine crucial for Treg function in homeostasis (30), EAE (36) and the curative T cell transfer colitis model (48). Furthermore, the inability of αv^−/−^ Treg to cure colitis was based on their negligible numbers in the inflamed colon, with Treg numbers being below a threshold that could confer immunosuppression. When a higher number of Treg was injected, leading to a relative increase in αv^−/−^ Treg found in the colon, Treg numbers appeared sufficient to suppress ongoing inflammation and mice recovered from colitis equally, whether αv^−/−^ or WT Treg had been injected. In summary, while definitive identification of the prevalent mechanism requires further investigation, data presented here suggest that αv integrins play an important role in Treg accumulation, but not function, specifically in the inflamed gut.

### αv Integrins Are Not Required for Treg Development and Immune Homeostasis

Despite the severe functional deficiency of Treg in the inflamed gut in the absence of αv, conditional Foxp3-αv^−/−^ mice were healthy and retained a normal Treg compartment in the steady state, including in the colon LP. This indicates that αv integrins are not required for Treg development, nor for homing and maintenance in the colon under homeostatic conditions. Global knockout of αv leads to early lethality in mice due to placental defects and defects in brain vasculogenesis leading to intracerebral hemorrhages (49). However, this phenotype is not due to lack of αv on lymphocytes, since conditional Lck-αv^−/−^ mice did not present with spontaneous autoimmune disease (25). We can further confirm that lack of αv integrins on Treg did not result in disturbed immune homeostasis in mice left to age up until 10 months of age. Therefore, αv integrin expression by Treg plays a far minor role in immune homeostasis than on DCs; mice lacking αvβ8 on DCs develop spontaneous colitis (25) due to the inability of CD103^+^ DCs to activate latent TGF-β and thereby induce adaptive Treg in the gut (26). We also found no role for αv integrins in Treg-mediated control of the initiation of an immune response, using an *in vitro* suppression assay, *in vivo* priming, and a preventive model of T cell transfer colitis. This highlights the context dependency of the functional relevance of Treg αv integrins. While αv^−/−^ Treg were capable of migrating to the colon in the preventive model of T cell transfer colitis, Treg are not needed in the colon to control disease in this model (5). Rather, Treg act on responder T cells in the secondary lymphoid organs. In contrast, in the curative model, Treg are required to migrate to the inflamed colon to resolve ongoing inflammation. Hence, αv integrins are dispensable for Treg to control T cell activation either in the steady or during the initiation of inflammation in secondary lymphoid organs, but are crucial to allow Treg to accumulate specifically in the inflamed gut and resolve inflammation.

Regulatory T cells appear not to be a homogenous population of cells with consistent regulatory functions. So-called “central” Treg are thought to represent a Treg pool responsible to keep homeostasis and control T cell activation in the secondary lymphoid organs. These have been characterized by expression of CCR7 and CD62L, directing their circulation through the lymphoid tissues (10, 17). On the other hand, so called “effector Treg” are deemed important in controlling inflammation in peripheral tissue, and among the markers attributed to this functional subgroup are KLRG1 and CD103 (18, 50, 51). Our study shows that αv integrins are important for the effector Treg function specifically in the context of the inflamed gut, but are redundant for the central Treg function of maintaining immune homeostasis.

### Active TGF-β Leads to Downregulation of αv Integrins on CD4^+^ T Cells

During iTreg generation, it was observed that αv surface expression was lost not only on CD4^+^ T cells from Foxp3-αv^−/−^ mice, but also on WT cells. This prompted further investigation and it was found that, in both conventional T cells and Treg, the presence of active TGF-β in the culture medium resulted in reduced surface αv levels, attributable at least in part to TGF-β signaling. Active TGF-β itself cannot bind to αv integrins, as the RGD tripeptide sequence that functions as integrin αv binding site is within the latency-associated peptide (52). It can therefore be excluded that observed reductions in αv levels are an artifact due to blocking of the binding site for the analysis antibody by TGF-β. It could be postulated that active TGF-β acts as a negative feedback regulator for αv integrin expression, since they have been implicated in the activation of latent TGF-β by a multitude of cell types (22). However, this finding stands in contrast to reports that αv integrins are upregulated by TGF-β signaling in several cell types including epithelial cells (53), keratinocytes (54) and fibroblasts (55, 56). While these divergent consequences of TGF-β signaling with respect to αv integrin expression deserve further investigation, they may well reflect the different biological contexts of TGF-β signaling in these cells.

### αvβ8 Is Redundant in Treg Function during Colitis

Regulatory T cells lacking integrin β8 showed no impaired capacity to reverse colitis. It is therefore notable that we could not confirm a reported crucial role for αvβ8, acting *via* activation of latent TGF-β, in this context (28). While we did not assess TGF-β activation or signaling, lack of a clinical deficiency when using β8-deficient Treg implies that this mechanism is redundant in this setting. Considering the high redundancy within αv integrins, with all αv integrins binding the RGD tripeptide sequence present in ECM components and latent TGF-β, a lack of αvβ8 may well be compensated by other αv integrins. We can only speculate on reasons for the discrepancy in results, despite having used the same curative model of T cell transfer colitis. Our findings are consistent with previous reports in that αvβ8-mediated TGF-β activation was not required for the prevention of T cell induced colitis (27, 28). It therefore appears that the requirement of αvβ8-mediated TGF-β activation is highly context-specific, and minor differences in experimental settings or the microbiota (57) of the utilized mice may be able to explain the contrasting results. Another report further supports this notion, showing that αvβ8 was not required in an oral tolerance model, despite the fact that Treg-derived TGF-β was crucial for the induction of tolerance in that model (58).

### αv Integrins Are Not Required for Treg Migration or Disease Resolution in EAE

A context-specific role for αv integrins is also highlighted in our own findings, since Foxp3-αv^−/−^ mice did not replicate the exacerbated and prolonged clinical course of EAE seen in WT mice depleted of Treg (35–37). While it is unclear whether Treg exert their function solely in the inflamed CNS or whether interactions with other immune cells are also important during the priming period in the periphery, it is evident from our data that αv integrins are not required for Treg to successfully migrate to the CNS and resolve disease. However, it cannot be ruled out with certainty that compensatory mechanisms may be masking a role for Treg αv integrins in the resolution of EAE; such mechanisms in effector T cells have previously been reported (23). It should be noted that WT Treg in the inflamed CNS exhibited high αv levels, as did WT Treg retrieved from the colon during a colitis model. The different functional implication of the loss of Treg αv integrins in these two models suggests that, at least in the case of αv, Treg change their expression pattern upon activation in a non-specific manner. This is also supported by the heightened αv expression levels in colonic Treg under steady state, which are known to display a more activated phenotype than their splenic counterparts (59). In effector T cells, this indeed appears to be the case, whereby αv was shown to be upregulated upon T cell activation in the lymph node, rather than at the site of inflammation (23). While an individual facet of these apparently global phenotypic changes seen among activated Treg may be critical to their function in one inflammatory setting, it can be entirely superfluous in another. This may be the basis of the high variability of reports assessing the functional relevance of specific gene expression changes in Treg, such as the highly debated role of T-bet expression in Treg function (8, 9, 12, 60, 61).

### Conclusion

In summary, our data provide evidence for a role of αv integrins in the successful accumulation of Treg in the inflamed colon, which is required for disease resolution. However, we did not identify αvβ8 as the integrin specifically required for resolution of colitis, indicating redundancy across the αv integrins. The superfluous role of αv integrins in Treg-dependent resolution of CNS inflammation highlights the necessity to unravel Treg mechanisms of action for individual diseases in order to target Treg successfully during inflammatory disease.

## Ethics Statement

This study was carried out in accordance with UK legislation. The protocol was approved by the University of Edinburgh Ethical Review Committee.

## Author Contributions

IM designed, performed, and analyzed experiments, interpreted the data, and wrote the article. SZ, IT, LS, RM, ML, and DS performed experiments and contributed to experimental design and methods. NH provided mice and helped to interpret the data. RO and SA designed experiments and assisted with data interpretation. SA contributed to writing the article. All authors revised the article.

## Conflict of Interest Statement

The authors declare that the research was conducted in the absence of any commercial or financial relationships that could be construed as a potential conflict of interest. The reviewer CS and handling editor declared their shared affiliation.

## Abbreviations

CNS, central nervous system; DC, dendritic cell; EAE, experimental autoimmune encephalomyelitis; iLN, inguinal lymph node; LP, lamina propria; mLN, mesenteric lymph node; pMOG, 35–55 peptide of mouse myelin oligodendrocyte glycoprotein; pOVA, ovalbumin peptide 323–339; Tn, naive CD4^+^ T cells; Treg, regulatory T cells; WT, wild-type.
