# Supplementary figures and images for: A Context-Dependent Role for αv Integrins in Regulatory T Cell Accumulation at Sites of Inflammation
Source: Front Immunol. 2018 Feb 26;9:264. doi: 10.3389/fimmu.2018.00264 (PMC5834440; doi:10.3389/fimmu.2018.00264)

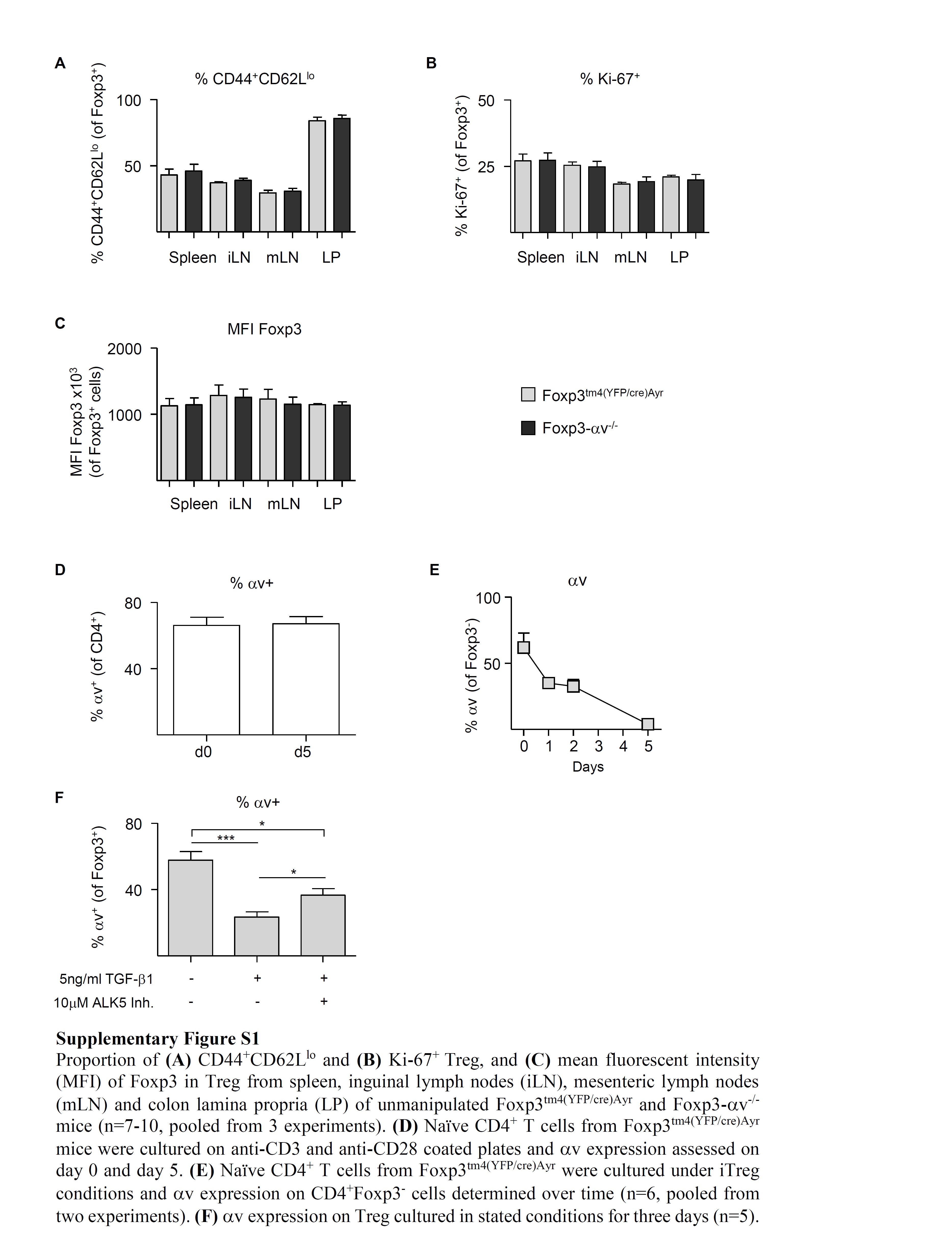

Supplement: Supplementary file 1 [file Image_1.jpeg]

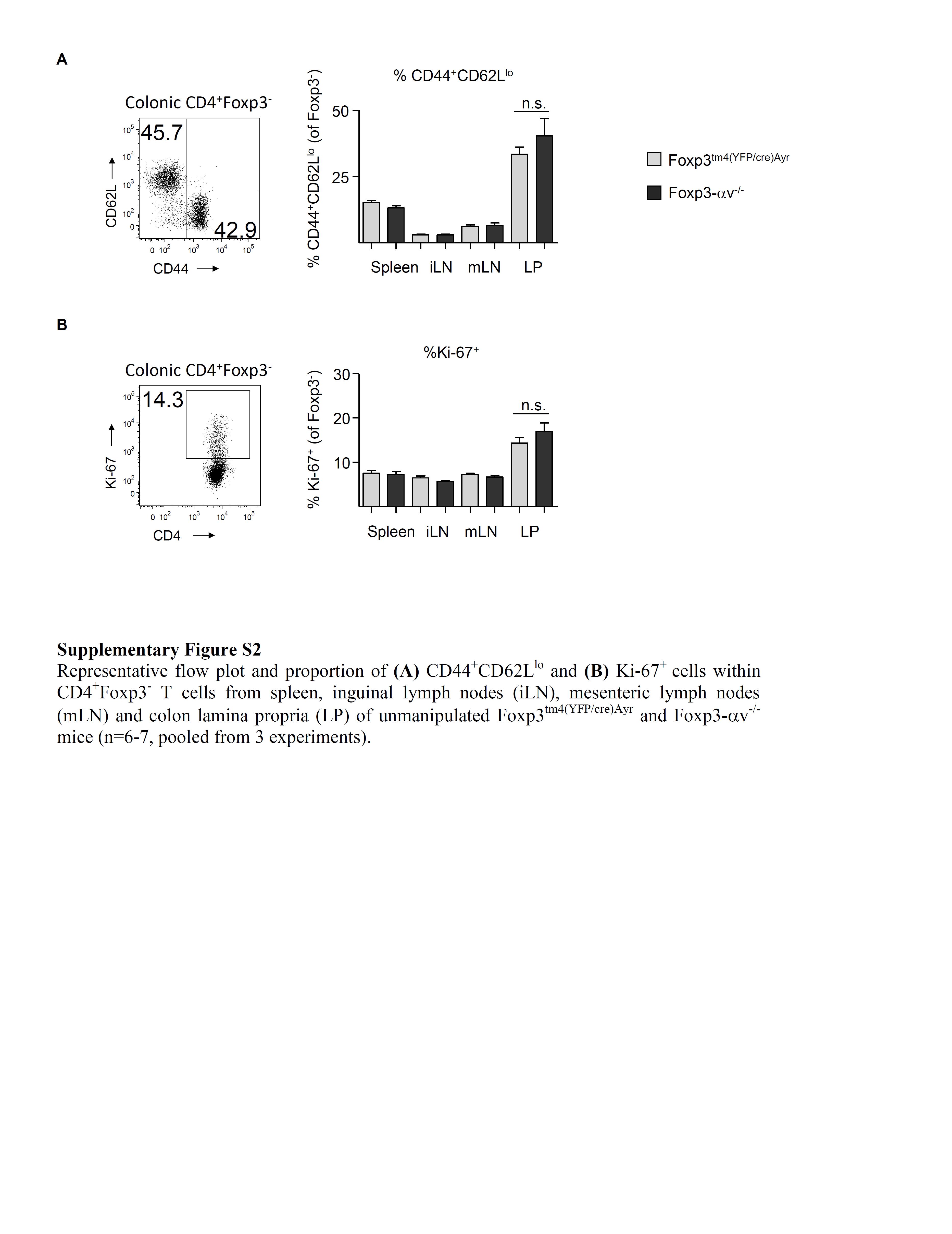

Supplement: Supplementary file 2 [file Image_2.jpeg]

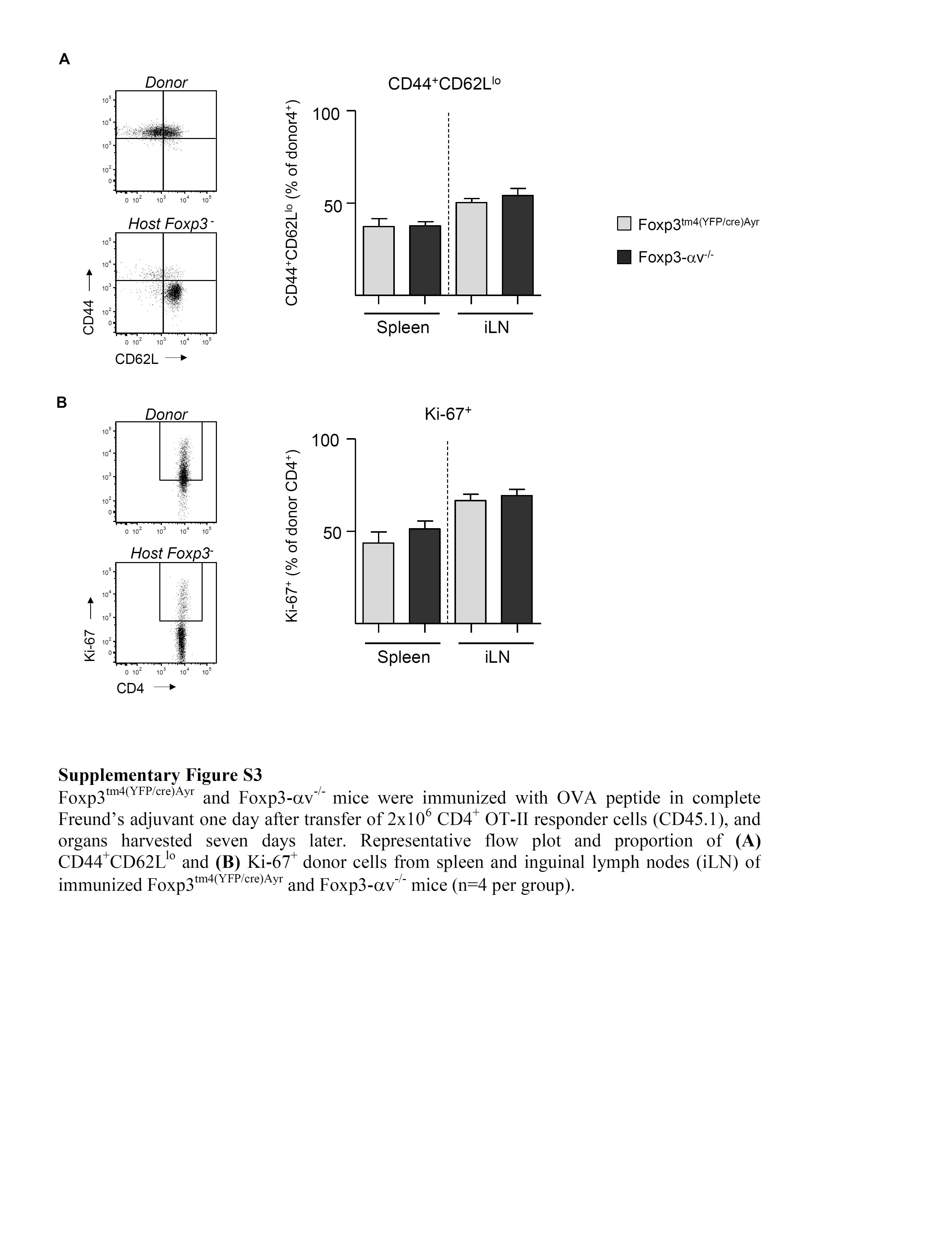

Supplement: Supplementary file 3 [file Image_3.jpeg]

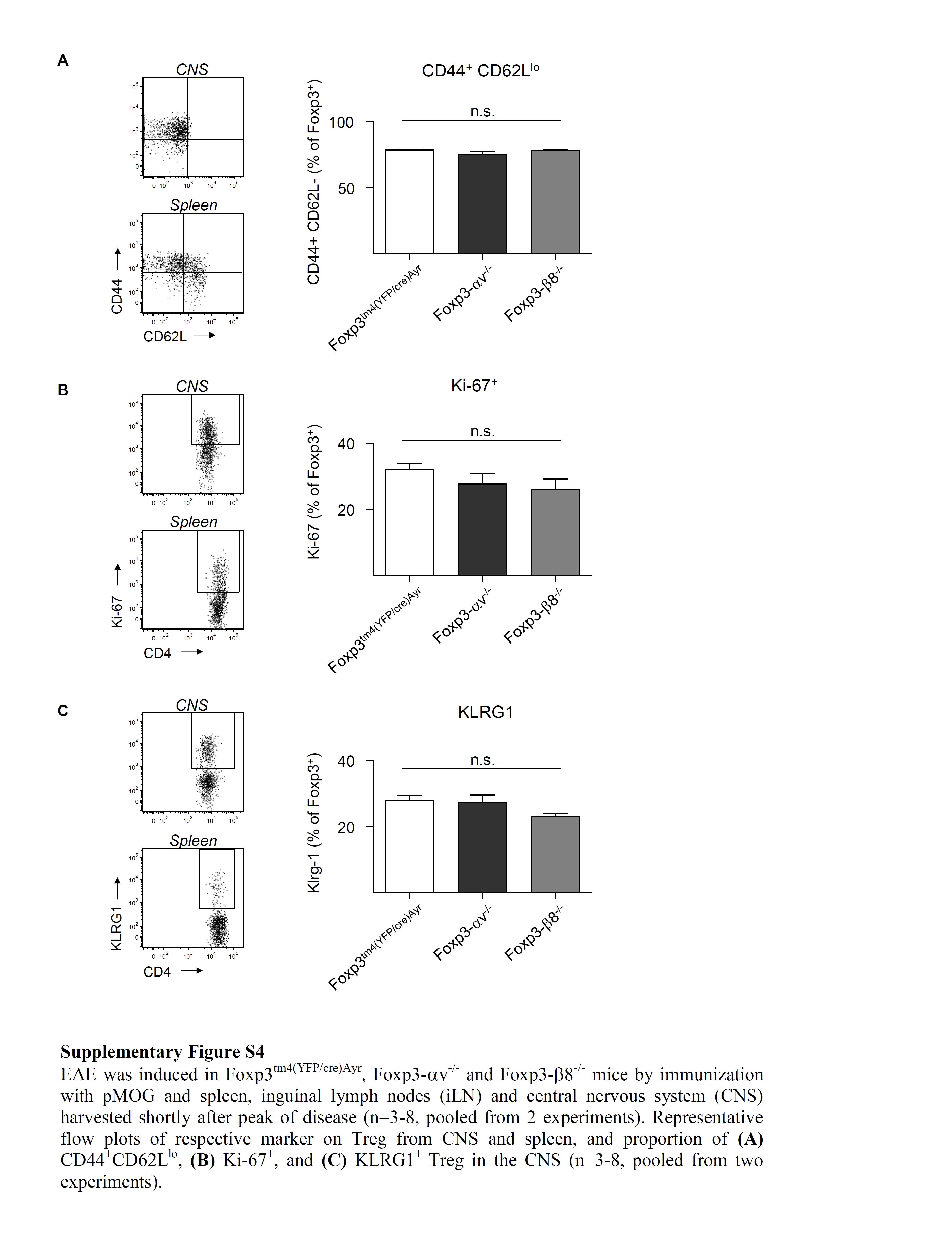

Supplement: Supplementary file 4 [file Image_4.jpeg]

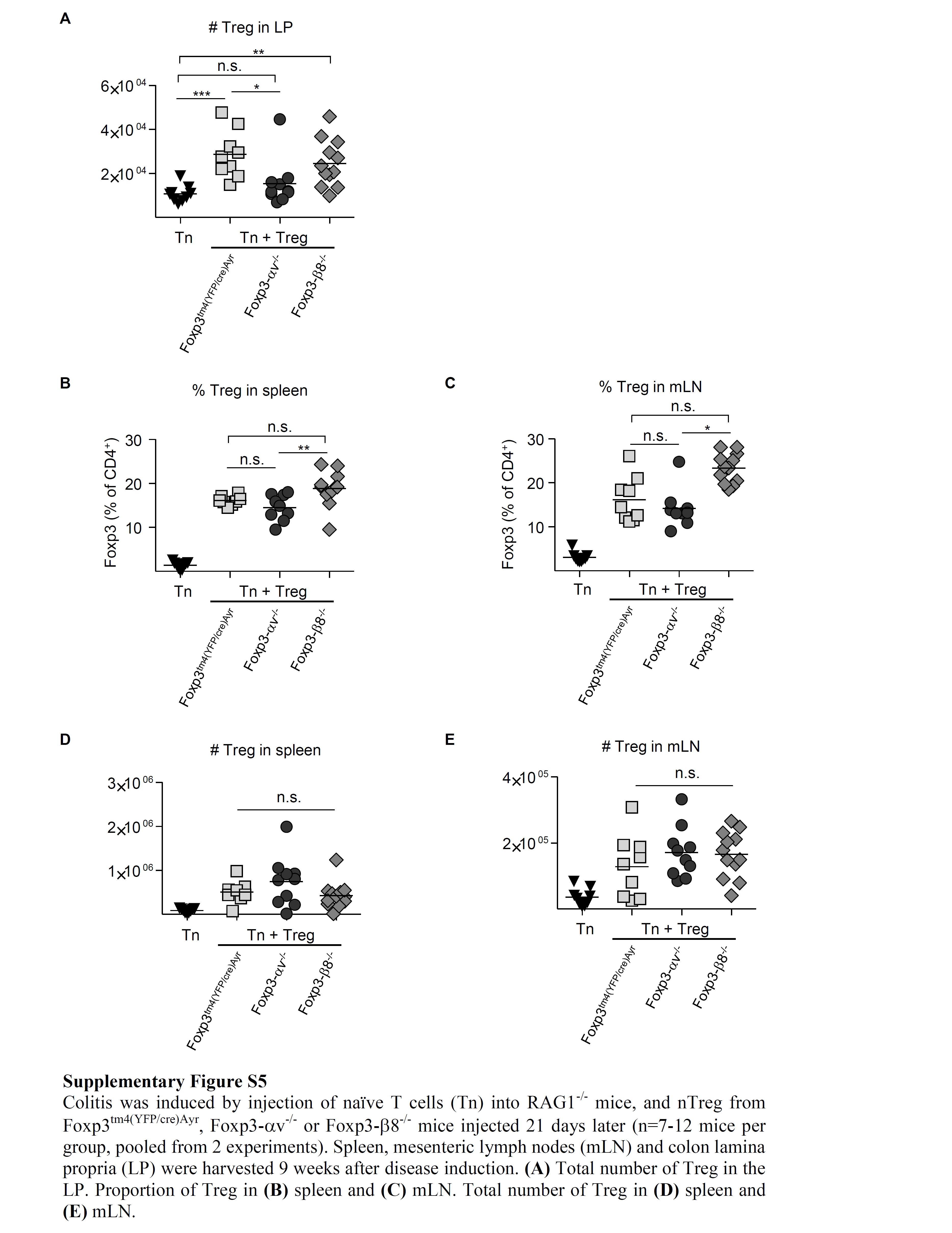

Supplement: Supplementary file 5 [file Image_5.jpeg]
